# Non-dystrophic myotonias: clinical and mutation spectrum of 70 German patients

**DOI:** 10.1007/s00415-020-10328-1

**Published:** 2020-12-02

**Authors:** Noemi Vereb, Federica Montagnese, Dieter Gläser, Benedikt Schoser

**Affiliations:** 1 Friedrich-Baur-Institute, Department of Neurology, Ludwig-Maximilians-University, Ziemssenstrasse 1a, 80336 Munich, Germany; 2 Genetikum, Neu-Ulm, Germany

**Keywords:** Non-dystrophic myotonia, Channelopathies, Myotonia congenita, *CLCN1*, *SCN4A*

## Abstract

**Introduction:**

Non-dystrophic myotonias (NDM) are heterogeneous diseases caused by mutations in *CLCN1* and *SCN4A*. The study aimed to describe the clinical and genetic spectrum of NDM in a large German cohort.

**Methods:**

We retrospectively identified all patients with genetically confirmed NDM diagnosed in our center. The following data were analyzed: demographics, family history, muscular features, cardiac involvement, CK, EMG, genotype, other tested genes, treatment perceived efficacy.

**Results:**

70 patients (age 40.2 years ± 14.9; 52.8% males) were included in our study (48 NDM-CLCN1, 22 NDM-SCN4A). The most frequent presenting symptoms were myotonia (NDM-CLCN1 83.3%, NDM-SCN4A 72.2%) and myalgia (NDM-CLCN1 57.4%, NDM-SCN4A 52.6%). Besides a more prominent facial involvement in NDM-SCN4A and cold-sensitivity in NDM-CLCN1, no other significant differences were observed between groups. Cardiac arrhythmia or conduction defects were documented in sixNDM-CLCN1 patients (three of them requiring a pacemaker) and one patient with NDM-SCN4A. CK was normal in 40% of patients. Myotonic runs in EMG were detected in 89.1% of CLCN1 and 78.9% of SCN4A. 50% of NDM-CLCN1 patients had the classic c.2680C>T (p.Arg894*) mutation. 12 new genetic variants are reported. About 50% of patients were not taking any anti-myotonic drug at the last follow-up. The anti-myotonic drugs with the best patient’s perceived efficacy were mexiletine and lamotrigine.

**Conclusion:**

This study highlights the relevant clinical overlap between NDM-CLCN1 and NDM-SCN4A patients and warrants the use of early and broad genetic investigation for the precise identification of the NDM subtype. Besides the clinical and genetic heterogeneity, the limited response to current anti-myotonic drugs constitutes a continuing challenge.

**Supplementary Information:**

The online version contains supplementary material available at 10.1007/s00415-020-10328-1.

## Introduction

Non-dystrophic myotonias (NDM) are rare hereditary neuromuscular diseases caused predominantly by mutations in *CLCN1* or *SCN4A*, respectively coding for the voltage-gated muscle channels ClC-1 and NaV1.4 [1, 2]. More than 150 *CLCN1* and at least 100 *SCN4A* myotonia-associated gene variants are currently known [3]. The common feature of these diseases is the altered electrical excitability of the muscular membrane [4]. In most cases, mutations lead to hyperexcitability, which manifests itself clinically as delayed muscle relaxation after voluntary contraction, also called myotonia [5–7]. The pathophysiological mechanism of myotonia is mainly related to reduced activity of the chloride channels and consequently reduced chloride conductivity in CLCN1, whereas in SCN4A-myotonias it is caused by an impaired inactivation of the NaV1.4 channels [5, 8].

CLCN1-myotonias, also known as myotonia congenita (MC), are to be distinguished into the autosomal dominant type Thomsen (TMC) and the autosomal recessive type Becker (BMC), related to the type of mutation. Although some CLCN1 mutations may be inherited both as autosomal recessive and dominant, making it difficult in some patients to classify them without a detailed genetic and clinical study of other family members. SCN4A-myotonias include paramyotonia congenita Eulenburg (PMC), potassium aggravated myotonia (PAM), and hyperkalemic periodic paralysis with myotonia (hyperPP) [9].

The phenotype of NDMs involves almost exclusively the skeletal muscle and, differently from dystrophic myotonias (DM), no relevant extra-muscular involvement has been described so far. The onset of symptoms is usually described in the first to the second decade of life; the lead symptom is myotonia, which is however often described by patients as stiffness, occurring especially during suddenly initiated movements after a resting period [9]. Otherwise, prolonged depolarization may temporarily reduce the excitability of the muscle membrane, causing transient weakness or periodic paralysis [8]. Significant clinical overlap exists between CLCN1 and SCN4A myotonias. Nevertheless, some features can help to distinguish both forms. CLCN1-myotonias are usually characterized by a warm-up phenomenon, worsening of myotonia under low temperature environment, and more prominent myotonia at lower limbs. Temporary weakness and segmental muscle hypertrophy occur mainly in patients with BMC [2, 10–12]. On the other hand, patients with PMC typically show worsening of their myotonia after repetitive movements, and under low-temperature environment. The SCN4A associated myotonia commonly involves the facial muscles with eyelid myotonia; the muscle weakness can occur more frequently and may persist longer than in CLCN1 patients [2, 11]. In addition to medical history and neurological examination, the detection of myotonic runs (MR) in electromyography (EMG) is an important hallmark for suspecting a NDM. Specific EMG protocols (repetitive stimulation, the “short” and “long” exercise tests, and the provocative cold test) even allow the differentiation between chloride and sodium channel myotonia with relatively high sensitivity and specificity [13]. Nevertheless, the precise diagnosis needs genetic analysis.

Up-to-date, no causal treatments are available for NDM but several drugs can be adopted for the symptomatic treatment of myotonia in the attempt to improve patients’ quality of life [2]. Presently, anti-myotonic drugs with the highest level of evidence concerning efficacy are mexiletine and lamotrigine [14–16]. Many other substances such as carbamazepine, flecainide, phenytoin, propafenone, or more recently cannabinoids are also being used [11, 17]. Unfortunately, many patients still do not report sufficient improvement of their core symptom myotonia.

Due to the rarity of these diseases, only a few large cohorts (*n* > 50) have been described in the literature to this date (Table 1). A description of a large genetically confirmed cohort of German NDM patients dates back to the year 1993 [18].

**Table 1:** Review of reported large NDM cohorts (> 50 patients)

Authors and year	*N* of pts				Patient’s origin	Main findings	Limitations
	Tot	CLCN1	SCN4A	Other			
Trivedi R. et al 2013 [12]	93^b^	32	34	9 (DM2) 17 (not genetically confirmed)	Caucasian and non-Hispanic	Prospective study Standardized assessments: clinic, laboratory, EMG, quality of life	Not only genetically confirmed NDM patients—mixed cohort
Statland, J.M. et al 2011 [23]	76^b^	27	25	5 (DM2) 19 (not genetically confirmed)	USA, Canada, UK	Usefulness of interactive voice response diary Weekly patients’ report on frequency of stiffness, pain, weakness, fatigue Stiffness most frequent and severe symptom in NDM No differences between SCN4A- and CLCN1-myotonia	Not only genetically confirmed NDM patients – mixed cohort Only patients’ reported symptoms
Trip, J. et al 2009 [24]	62^c^	32	30	–	The Netherlands	Description of similarities and differences between NDM-CLCN1 and NDM-SCN4A patients Development of clinical guideline for genetic testing Robust methodology	Lack of laboratory and EMG data
Dupré N. et al. 2009 [22]	50	36	14	–	French-Canadian	Genotype–phenotype correlations p.M485V as most common *CLCN1* mutation Therapy mostly needed in recessive CLCN1-myotonia	Few SCN4A patients
Trip, J. et al 2008 [25]	54^c^	32	22	–	The Netherlands	Tandem analysis of *CLCN1* and *SCN4A* p.F413C most common *CLCN1* mutation 13 novel mutations in *CLCN1* p.G1306V most common in *SCN4A* 3 novel mutations in *SCN4A*	3 recessive and three sporadic patients with no second mutation found
Baumann P. et al. 1998 [21]	54^a^	–	–	–	Finland	High incidence of MC in Finland Mean age at onset 11 years Mean diagnostic delay 18 years	Lack of genetic confirmation
Becker P.E. 1977 [27]	146^a^	-	–	–	Germany	Clinical and EMG characterization of patients	Lack of genetic confirmation
Koch M.C. 1993 [18]	78	78			Germany	Clinical and EMG characterization of 37 patients of 14 families with 78 patients Linkage analysis and Sanger sequencing of *CLCN1*	First genetically proven German myotonia congenita cohort
Sansone V.A. et al. 2012 [26]	488	36	26	382 (DM1) 40 (DM2) 4 (Andersen-Tawil)	Italy	Skeletal muscle channelopathies have a similar Individualized Quality of Life-index as DMs Patients with Myotonia congenita are especially affected by myotonia Pain is present in patients with myotonia congenita, hypoPP and DM2, but not in DM1	Results obtained exclusively from patient questionnaires

*NDM* non-dystrophic myotonia

^a^Not genetically confirmed

^b,c^Same cohort of patients

The aim of this study was to investigate the clinical features of a large cohort of German patients with NDM, highlighting some diagnostic and therapeutic challenges and expanding the genotype by describing newly detected genetic variants.

## Patients and methods

### Patients

This is a monocentric, retrospective study, performed at the Friedrich-Baur-Institute, Munich, Germany. We have included patients of all ages that, according to our institutional database of patient records, had a genetically confirmed diagnosis of NDM. If new genetic variants were detected, their pathogenicity was evaluated considering the presence of positive family history, documented typical clinical signs or symptoms of NDM, and the presence of myotonic runs in EMG. The database search refers to data archived between the years 1994 to 2019. Additional clinical data were obtained by additionally reviewing paper records of the identified patients.

### Methods

The following data were collected and analyzed: demographics, age at onset, family history, first symptom, symptoms present during disease progression (myotonia, weakness, muscle hypertrophy and atrophy, myalgia, cramps, dysphagia, dysphonia), symptoms at the last follow-up visit, presence of any type of cardiac involvement, creatine kinase (CK), EMG, genetic results including previously tested genes, diagnostic delay (time between the onset of symptoms and the genetic diagnosis), past and current anti-myotonic medications with patients perceived efficacy.

For disease onset, either the exact year or the development stage (e.g., birth, infancy, childhood…) were collected according to the information available from patient’ records. The age groups were determined as follows: < 1 year as infant, 1–9 years as child, 10–17 years as adolescent, 18–35 years as young adult, 36–55 years as middle-aged adult, and > 55 years as old adult [19, 20]. We combined family history, clinical features, EMG, and genetic results to classify patients with CLCN1 mutations in either Becker (BMC) or Thomsen myotonia (TMC).

The following cardiac abnormalities were included: conduction defects, cardiac arrhythmia, or cardiomyopathies.

Due to various reference values of CK through the years, all values were expressed as the percentage of normal value and converted into absolute values considering gender-specific reference values of our laboratory. The CK is reported referring to the highest CK values found. The EMG data refer to the first documented EMG. The efficacy of anti-myotonic treatment was semiquantitatively evaluated as 1 = excellent, 2 = good, or 3 = poor according to the patient evaluation reported in patient records by the treating doctor. The follow-up period was calculated between the first and the last recorded follow-up visit.

### Statistic analysis

All statistical analyses were performed with IBM SPSS Statistics (Version 25.0.0.1). The normality of variables was assessed by Shapiro–Wilk-test. Descriptive analysis included mean ± standard deviation (SD) or median and interquartile range (IQR), as appropriate, for continuous variables. Frequencies were calculated for categorical variables. For normally distributed continuous variables unpaired Student’s tests were used to assess differences between groups, while for non-normally distributed continuous variables the Mann–Whitney *U* test was used. *χ*^2^ or Fisher’s exact test was used to compare frequencies. All hypothesis tests conducted were two-tailed. A *p* value < 0.05 was considered significant.

## Results

### Clinical features

**Fig. 1:**
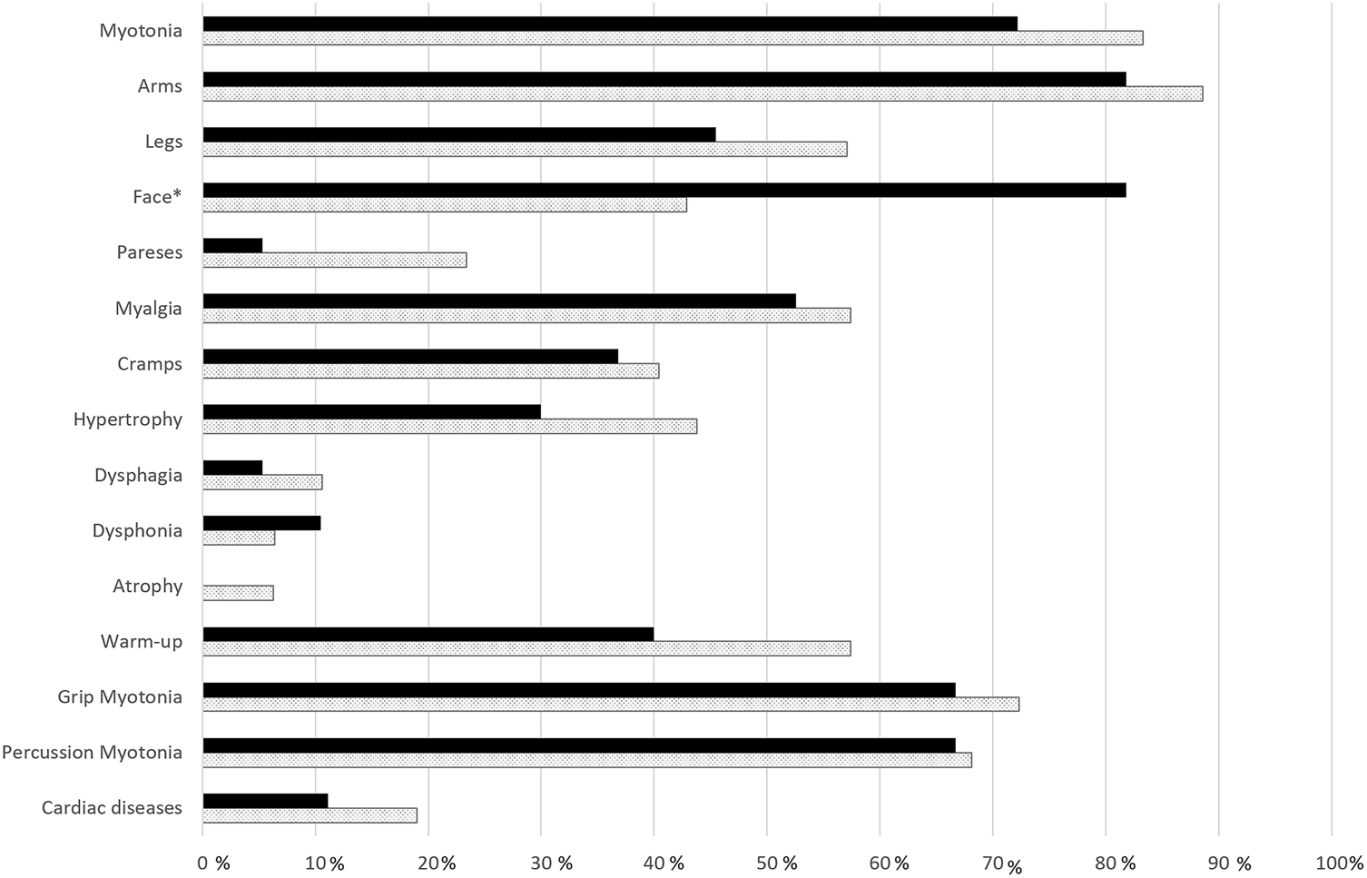
Comparison of symptoms in patients with NDM-CLCN1 and NDM-SCN4A. *Statistically significant; filled: CLCN1; Dotted: SCN4A—(created with Excel)

We identified 76 patients with clinical suspicion of NDM and genetic results. Six patients were excluded due to inconclusive genetic results or lack of sufficient clinical data. We enrolled 70 patients (64 families): 48 (68.5%) patients had a diagnosis of CLCN1-myotonia and 22 (31.4%) of SCN4A-myotonia. The mean age was 40.2 ± 14.9 years, with no significant difference between the two groups. Thirty-seven patients were male (52.8%). No gender differences were identified for any of the analyzed variables. A detailed summary of the core clinical features of this cohort and the differences between NDM-CLCN1 and NDM-SCN4A are shown in Table 2 and Fig. 1.

**Table 2:** Demographics and clinical features of this cohort of patients

Variable	*N*	NDM-CLCN1	NDM-SCN4A	*p* value	CLCN1-AD (TMC) *n* = 12	CLCN1-AR (BMC) *n* = 28	*N*	*p* value
Age	70	40.91 ± 15.05 years	38.70 ± 14.82 years	0.57	47.7 ± 13.9	46.8 ± 16.08	40	0.91
Gender	70	52.08% (m), 47.92% (f)	54.55% (m), 45.45% (f)	0.85	58.3% (m)	53.6% (m)	40	0.82
Family history	66	50.0% pos., 50.0% neg	70.0% pos., 30.0% neg	0.11	–	–		
Mean follow-up	70	4.83 ± 6.74 years	3.20 ± 4.15 years	0.92	7.3 ± 6.45	3.2 ± 6.9	40	0.08*
Age at onset	39	13.50 (10.00–29.50)	14.00 (10.00–40.00)	0.54	19.2 (10–37.5)	16.9 (8.7–25.5)	23	0.66
Symptoms at onset:							34	
Myotonia	62	85.7%	50%	0.003*	90.9%	86.9%		0.47
Myalgia	62	19.0%	10.0%	0.48	36.3%	4.3%		0.029*
Weakness/paresis	62	11.9%	20.0%	0.45	18%	13%		0.52
Cramps	62	9.5%	10.0%	1.00	–	–		
Periodic paralysis	62	0.0%	20.0%	0.009*	–	–		
Symptoms during follow-up							40	
Myotonia	66	83.3%	72.2%	0.32	66.6%	92.8%		0.048*
Paresis	66	23.4%	5.3%	0.16	25%	25.9%		1
Myalgia	66	57.4%	52.6%	0.72	66.6%	48.1%		0.32
Cramps	66	40.4%	36.8%	0.79	–	–		
Muscle hypertrophy	68	43.8%	30.0%	0.42	16.6%	42.8%		0.15
Dysphagia	66	10.6%	5.3%	0.66	–	–		
Dysphonia	66	6.4%	10.5%	0.62	–	–		
Muscle atrophy	67	6.3%	0.0%	0.55	–	–		
Myotonia localisation							35	
Arms	46	88.6%	81.8%	0.56	100.00%	86.3%		0.37
Legs	46	57.1%	45.5%	0.73	25%	68.18%		0.045*
Face	46	42.9%	81.8%	0.024*	25%	50%		0.4
Myotonia characteristics							39	
Warm-up	62	57.4%	40.0%	0.24	50%	66%		0.47
Grip myotonia	62	72.3%	66.7%	0.75	58.3%	74%		0.45
Percussion myotonia	62	68.1%	66.7%	1.00	75%	77.7%		1
Triggers							21	
Cold	41	89.3%	46.2%	0.005*	88.8%	100%		0.42
Psychological stress	41	14.3%	7.7%	1.00	–	–		
Occurrence of infections	41	0.0%	7.7%	0.32	–	–		
Exercise	41	3.6%	0.0%	1.00	0%	6.2%		0.58
Alcohol consumption	41	3.6%	0.0%	1.00	–	–		
Potassium exposure	41	0.0%	14.3%	0.11	–	–		
Cardiac involvement								
Cardiac involvement (tot)	38	19%	11.1%	0.31	18.18%	44.44%	35	0.23
Diagnostic assessments							38	
Diagnostic delay	37	17.23 ± 11.93 years	15.27 ± 13.47 years	0.66	18.5 ± 9.7	17.8 ± 12.3		0.88
Elevated CK	66	40.5%	38.1%	1.00	58.82%	60.00%		0.93
Minimum CK	30	291.00 U/l (222.0–433.3)	251.40 U/l (197.3–607.7)	0.63				
Maximum CK	38	286.01 U/l (226.5–467.7)	373.67 U/l (213.4–1106.8)	0.59				
EMG							38	
Myotonic runs	65	89.1%	78.9%	0.43	90.9%	100%		0.29
Complex-repetitive discharges	65	4.3%	10.5%	0.57				
Other pathological spontaneous activity	65	13.0%	15.8%	0.71				

*Statistically significant

The onset of symptoms was reported mostly in childhood (39.6%) or adolescence (28.5%) and no patient reported the onset of symptoms after the fifth decade. No statistically significant difference was observed in age at onset between NDM-CLCN1 and NDM-SCN4A. The most frequent symptom at onset was myotonia (74.1%), followed by myalgia (16.1%) and muscle weakness (14.5%). Myotonia at onset was more frequent in patients with CLCN1-myotonia (85.7%) than in patients with SCN4A-myotonia (50%) (*p* = 0.003); periodic paralysis was only reported in patients with SCN4A-myotonia (20%) (*p* = 0.008).

At the last documented follow-up visit, the majority of patients presented myotonia (NDM-CLCN1 83.3%, NDM-SCN4A 72.2%, *p* = 0.31) and up to 25% of patients presented muscle weakness at their final neurological examination (NDM-CLCN1 23.4%, NDM-SCN4A 5.2%, *p* = 0.15). We could not find any statistically significant changes of manual muscle testing between the first and last examinations (*p* = 0.184). The distribution of myotonia is reported in Table 2, facial involvement was significantly more frequent in patients with SCN4A-myotonia (81.8% vs. 42.8%, *p* = 0.024). Cold temperature environment was the most common trigger for myotonia in all patients (75.61%), being however more frequently reported as a factor triggering or worsening symptoms from NDM-CLCN1 patients (89.2% vs. 46.1%, *p* = 0.005).

More than half of the patients reported myalgia during the disease course (NDM-CLCN1 57.4%, NDM-SCN4A 52.6%, *p* = 0.72). Additionally, about 30–40% of patients reported muscle cramps with no significant differences between both types. Dysphagia and dysphonia were only rarely reported and mostly mild. Muscle hypertrophy was documented in both subtypes (NDM-CLCN1 43.7%, NDM-SCN4A 30%, *p* = 0.41), whereas muscle atrophy was only documented in a few NDM-CLCN1-patients (6.2%).

In the NDM-CLCN1 cohort, we classified 28 patients as BMC, 12 patients as TMC and in 8 cases it was not possible to clearly classify them as BMC or TMC. Comparing the features of BMC and TMC we found significant differences only as regards the presence of myalgia (more common in TMC), the presence of myotonia (more prevalent in BMC), and the distribution of myotonia, more frequently occurring in the legs of BMC patients (Table 2). Cardiac involvement was reported in six patients with NDM-CLCN1 and one patient with NDM-SCN4A (Table 2). A detailed description of the cardiac pathologies is reported in Table 3. Six NDM-CLCN1 patients had relevant cardiac arrhythmias, in one with an early onset before age 45. Of these, three required pacemaker implantation.

**Table 3:** Cardiac involvement

Pt. ID	Gender	Age	NDM	Onset of cardiac involvement	Cardiac involvement	Mutation 1	Mutation 2
8	M	56	CLCN1	54	Complete right bundle branch block	c.2680C>T (p.Arg894*)	–
10	F	48	CLCN1	45	Cardiac arrhythmia	c.870C>G (p.Ile290Met)	–
17	M	68	SCN4A	Not known	Mild cardiac arrhythmia	c.1333G>A (p.Val445Met)	–
**29**	**F**	**78**	CLCN1	**Not known**	**Global heart failure and pacemaker implantation unknown cause**	c.2680C>T (p.Arg894*)	c. 1238 T>G (p.Phe413Cys)
32	M	56	CLCN1	Not known	Cardiac arrhythmia, AV-block I°, incomplete right bundle branch block	c.2680C>T (p.Arg894*)	c.696G>A (p.Glu232Glu)
**41**	**F**	**54**	**CLCN1**	**50**	**AV-block III°, pacemaker implantation**	c.313C>T (p.Arg105Cys)	c.501C>G (p.Phe167Leu)
**71**	**F**	**40**	CLCN1	**30**	**AV-block II° and syncope, pacemaker implantation**	c.1649C>G (p.Thr550Arg)	c.1649C>G (p.Thr550Arg)

Bold = cases of interest

*AV-Block* atrioventricular block

### Diagnostic assessments

The family history was positive in 56% of patients (50% NDM-CLCN1, 70% NDM-SCN4A patients). The mean diagnostic delay was 16.6 ± 12.2 years, without significant difference between both types or between patients with or without positive family history for myotonia. Furthermore, there was no significant difference in diagnostic delay between patients who were genetically tested after the year 2010 (16 ± 11.9 years) and those before (19.1 ± 14.2 years) (*p* = 0.55).

40.5% of NDM-CLCN1 and 38.1% of NDM-SCN4A patients had normal CK values. In patients with elevated CK, the CK was on average 2–3 times upper normal limit with no relevant differences in both groups (median and IQR in NDM-CLCN1: 286.0 U/L, 226.5–467.7 U/L; median and IQR in NDM-SCN4A: 373.7 U/L, 213.4–1106.8 U/L).

The time between the onset of the disease and the performance of the first EMG was on average 12.6 ± 10.7 years, with no significant differences between NDM-CLCN1 and NDM-SCN4A (14.2 ± 11.4 years for NDM-CLCN1 and 8.7 ± 7.9 years in NDM-SCN4A; *p* = 0.152). EMG was normal in only 7.7% of the patients. Myotonic runs were detected in 86.2% of patients in their first documented EMG, other forms of pathological spontaneous activity as fibrillation potentials and pseudo-myotonic runs were present in 18.5% of the patients.

A genetic confirmation was obtained in all patients. In 24/70 no additional genes were tested before reaching the correct diagnosis (Supplementary Table 1). In the remaining 46/70 patients, a tandem analysis of *SCN4A* and *CLCN1* was performed in 15.2% and next generation sequencing techniques were adopted in 58.9% of cases. In addition to other genes responsible for NDM, the most common differential diagnosis was myotonic dystrophy, investigated in 47.1% of patients.

The genotype of all patients is shown in Table 4. Half of NDM-CLCN1-myotonia patients (*n* = 24) had at least one heterozygous c.2680C>T mutation. The most common mutations in SCN4A were the c.3917G>T (*n* = 5), c. 1333G>A (*n* = 4) and c.2111C>T (*n* = 3). A total of 12 new genetic variants could be identified (highlighted in bold in Table 3). Newly identified mutations included frameshift, in-frame, missense, and splice-site mutations. The patients with novel variants did not show peculiar clinical features different from those of other patients with known CLCN1 or SCN4A mutations.

**Table 4:** Mutational spectrum

Pt. ID	Gene	Inheritance in the family*	Mutation 1	Mutation 1	Mutation 2	Mutation 2
1	CLCN1	AR	c.407A>G	p. Asp136Gly	n.f	n.f
3	CLCN1	AR	c.1437_1450del	p.Pro480Hisfs*24	**c.2422_2427dup**	**p.Glu808_Gln809dup**
4	CLCN1	AD	c.2680C>T	p.Arg894*	c.2680C>T	p.Arg894*
5	CLCN1	AD/AR	c.937G>A	p.Ala313Thr	c.2680C>T	p.Arg894*
6	CLCN1	AR	c.1437_1450del	p.Pro480Hisfs*24	c.2680C>T	p.Arg894*
8	CLCN1	AD	c.2680C>T	p.Arg894*		
9	CLCN1	AR	c.1444G>A	p. Gly482Arg	n.f	n.f
10	CLCN1	AD	c.870C>G	p.Ile290Met		
12	CLCN1	AR	c.2680C>T	p.Arg894*	c.180+3A>T	p.Gln60ins*22
13	CLCN1	AR	c.2680C>T	p.Arg894*	c.2680C>T	p.Arg894*
20	CLCN1	AR	c.1013G>A	p.Arg338Gln	c.1478C>A	p.Ala493Glu
22	CLCN1	AR	c.180+3A>T	p.Gln60ins*22	c.180+3A>T	p.Gln60ins*22
27	CLCN1	AD	c.2680C>T	p.Arg894*	**c.2564G**>**A**	**p.Gly855Glu**
29	CLCN1	AR	c.2680C>T	p.Arg894*	c.1238T>G	p.Phe413Cys
30	CLCN1	AD	c.2680C>T	p.Arg894*		
31	CLCN1	AD	c.937G>A	p.Ala313Thr		
32	CLCN1	AR	c.2680C>T	p.Arg894*	**c.696G**>**A**	**p.Glu232Glu**
33	CLCN1	AD	c.2680C>T	p.Arg894*		
34	CLCN1	AD	**c.568G**>**A**	**p.Gly190Arg**		
35	CLCN1	AR	c.180+3A>T	p.Gln60ins*22	c.2680C>T	p.Arg894*
36	CLCN1	AR	c.2680C>T	p.Arg894*	c.2680C>T	p.Arg894*
39	CLCN1	AR	c.501C>G	p.Phe167Leu	c.1437_1450del	p.Ile479Ilefs*25
41	CLCN1	AR	c.313C>T	p.Arg105Cys	c.501C>G	p.Phe167Leu
42	CLCN1	AD	**c. 983C**>**T**	**p.Thr328Ile**		
43	CLCN1	AR	c.2401G>T	p.Glu801*	**c.613G**>**A**	**p.Glu205Lys**
45	CLCN1	AR	**c.2114_2115het_insT**	**p.Pro705ProfsX8**		
46	CLCN1	AR	c.2926C>T	p.Arg976X	c.1655A>G	p.Gln552Arg
47	CLCN1	AR	c.2680C>T	p.Arg894*	**c.1444_1449delGGAGGC**	**p.Gly482_Gly483del**
48	CLCN1	AD	c.2680C>T	p.Arg894*		
49	CLCN1	AD	c.2680C>T	p.Arg894*		
52	CLCN1	AD	**c.1445G**>**A**	**p.Gly482Glu**		
53	CLCN1	AR	c.2680C>T	p.Arg894*	c.2680C>T	p.Arg894*
54	CLCN1	AD	c.937G>A	p.Ala313Thr		
55	CLCN1	AR	c.180+3A>T	p.Gln60ins*22	c.2680C>T	p.Arg894*
56	CLCN1	AR	c.180+3A>T	p.Gln60ins*22	c.1488G>T	p.Arg496Ser
57	CLCN1	AR	c.568G>A	p.Gly190Arg	**c.1166**+**5G**>**A**	**p.?**
58	CLCN1	AR	c.1238T>G	p.Phe413Cys	n.f	n.f
60	CLCN1	AD	c.1655A>G	p.Gln552Arg		
62	CLCN1	AD	c.2680C>T	p.Arg894*		
63	CLCN1	AR	c.2680C>T	p.Arg894*	c.1013G>A	p.Arg338Gln
64	CLCN1	AD	c.2680C>T	p.Arg894*		
65	CLCN1	AR	c.979G>A	p.Val327Ile	c.1437_1450del	p.Pro480Hisfs*24
66	CLCN1	AR	c.2680C>T	p.Arg894*	c.2680C>T	p.Arg894*
67	CLCN1	AD	c.929C>T	p.Thr310Met		
70	CLCN1	AD	c.2680C>T	p.Arg894*		
71	CLCN1	AR	c.1649C>G	p.Thr550Arg	c.1649C>G	p.Thr550Arg
72	CLCN1	AR	c.180+3A>T	p.Gln60ins*22	c.1488G>T	p.Arg496Ser
73	CLCN1	AR	c.2680C>T	p.Arg894*	c.2680C>T	p.Arg894*
2	SCN4A	AD	c.2024G>A	p. Arg675Gln		
7	SCN4A	AD	c.2111C>T	p.Thr704Met		
11	SCN4A	AD	**c.4106C**>**A**	**p.Thr1369Asn**		
14	SCN4A	AD	c.3917G>C	p.Gly1306Ala		
15	SCN4A	AD	**c.1333G**>**T**	**p.Val445Leu**		
16	SCN4A	AD	c.2078T>C	p.Ile693Thr		
17	SCN4A	AD	c.1333G>A	p.Val445Met		
18	SCN4A	AD	c.1333G>A	p.Val445Met		
19	SCN4A	AD	c.1333G>A	p.Val445Met		
21	SCN4A	AD	c.3917G>T	p.Gly1306Val	c.3917G>T	p.Gly1306Val
23	SCN4A	AD	c.5113T>A	p.Phe1705Ile		
24	SCN4A	AD	c.2111C>T	p.Thr704Met		
26	SCN4A	AD	c.3917G>C	p.Gly1306Ala		
28	SCN4A	AD	c.3877G>A	p.Val1293Ile		
37	SCN4A	AD	c.4342C>T	p.Arg1448Cys		
40	SCN4A	AD	c.1333G>A	p.Val445Met		
51	SCN4A	AD	c.3893T>G	p.Phe1298Cys		
61	SCN4A	AD	c.3917G>T	p.Gly1306Val		
69	SCN4A	AD	c.2111C>T	p.Thr704Met		
74	SCN4A	AD	c.3917G>T	p.Gly1306Val		
75	SCN4A	AD	c.3917G>T	p.Gly1306Val		
76	SCN4A	AD	c.3917G>T	p.Gly1306Val		

*Some mutations in the *CLCN1* gene have both a dominant or a recessive effect (e.g., p.R894*, p.A313T)

### Treatment

The mean follow-up duration for treatment was 4.3 ± 6 years, with no significant differences between patients with NDM-CLCN1 or NDM-SCN4A (*p* = 0.92). From the review of all patients’ records, 13.3% of patients have never taken an anti-myotonic drug (11.4% of CLCN1 and 20% of SCN4A, *p* = 0.6). At the last follow-up visit, 48.9% of patients (44.7% of NDM-CLCN1, 63.6% NDM-SCN4A) were not taking any anti-myotonic medication, either because the symptoms’ burden was too low or because of the low efficacy or side effects of the drugs tested. All patients being treated with anti-myotonic drugs (51%) received a monotherapy. On average, two different anti-myotonic drugs were tested in the past (range 1–5). A list of the reported medications is provided in Table 5, including the level of patients’ perceived efficacy, documented by the treating physician.

**Table 5:** Anti-myotonic treatment in NDMs

Drug	Dose (mg)	*N* (total)	NDM-CLCN1%	NDM-SCN4A%	Patients’ perceived efficacy (mean value score)		
					*n*	NDM-CLCN1	NDM-SCN4A
Commonly used anti-myotonic drugs							
Carbamazepine	100–1600	19	78.9	21.1	12	2.9	3
Flecainide	50–300	16	87.5	12.5	14	2.5	3
Mexiletine^a^	200-600^b^	15	80	20	10	1.89	2
Lamotrigine^a^	50–300	8	75	25	6	2.25	2
Acetazolamide	125–500	6	33.3	66.7	3	n.a	2.3
Phenytoin	100–300	5	80	20	3	3	3
Mexiletine ret.^a^	360–720	4	100	0	4	1.3	1
Propafenone	150–450	2	100	0	2	3	n.a
Other drugs with potential anti-myotonic effect							
Tolperisone	50–450	18	83.3	16.7	13	2.9	3
Gabapentin	100–3600	11	72.7	27.3	8	2.8	2.7
Methocarbamol	750–4500	9	77.8	22.2	8	2.8	2.5
Pregabalin	150–600	5	80	20	n.a	n.a	n.a
Baclofen	10–75	2	100	0	n.a	n.a	n.a
Cannabinoid/THC	20.58–41.16/2.2–6.02	2	100	0	2	2	n.a
Oxcarbazepine	35–150	1	100	0	n.a	n.a	n.a

^a^Anti-myotonic drugs with perceived best efficacy; 1 = excellent, 2 = good, 3 = poor

^b^One patient received up to 1200 mg with major side effects

## Discussion

In this study, we depicted the clinical features of a large cohort of German patients with NDM thus adding valuable information to the few reported large cohorts of patients (Table 1) [12, 18, 21–27]. The core clinical features of our NDM cohort were mostly confirmatory of literature data, highlighting the significant clinical overlap between patients with NDM-CLCN1 and NDM-SCN4A. However, some new findings could be summarized. The age of onset could be collocated in the 1st to 2nd decade of life in most patients. Myotonia was the most prominent symptom, occurring more frequently in facial muscles in NDM-SCN4A and not significantly but tendentially more frequently in the legs for NDM-CLCN1, muscle weakness tended to occur more frequently in NDM-CLCN1. Myalgia was, immediately after myotonia, the most prevalent symptom during disease progression involving 52–57% of patients, without significant differences between NDM-CLCN1 and NDM-SCN4A. In the literature, the prevalence of pain in NDMs widely ranges from 28 to 53% in NDM-CLCN1 and from 56 to 82% in NDM-SCN4A [12, 23, 24]. Some *SCN4A* mutations are known to be associated with myalgia (e.g., c.1333G>A, c.3917G>C, c.3466G>A), in our patients only 6/22 showed either c.1333G>A or the c.3917G>C mutation, thus explaining only a minority of myalgic patients. The presence of pain is one of the major determinants of low quality of life in NDM patients [28] and needs to be properly monitored and treated. Some myotonia characteristics are considered more archetypal for one form or the other, as warm-up phenomenon and muscle hypertrophy usually points toward NDM-CLCN1 and cold sensitivity toward NDM-SCN4A. In contrast, in our genetically confirmed cohort a more relevant clinical overlap was observed as the muscle hypertrophy, the warm-up phenomenon as well as grip- and percussion myotonia were frequently observed in both groups. Even the classic triggers were present in both NDM groups with an even higher prevalence of cold sensitivity for NDM-CLCN1 patients. This finding is untypical as usually NDM-SCN4A patients refer a more prominent worsening of symptoms with cold temperatures, but it is well known that cold may worsen symptoms also in NDM-CLCN1 patients. Similar observations were also reported by Trivedi et al. [12] and should favor a genetic tandem analysis approach rather than a single gene sequencing. Extra-muscular manifestations are thought to be non-typical for NDM patients, even though the role of *SCN4A* variants in causing cardiac arrhythmias and Brugada syndrome remains debated [29]. In our cohort, no patient with *SCN4A* mutations presented relevant cardiac involvement; on the other hand, six NDM-CLCN1 patients had cardiac arrhythmias or conduction defects, requiring the implantation of a pacemaker in three patients. In particular, patient 65 required pacemaker implantation at age 30 years and has a positive family history of dilative cardiomyopathy documented in her father’s and brother’s medical history, the latter also presented with a NDM-CLCN1-myotonia. Given the autosomal recessive nature of the two known pathogenic *CLCN1* gene mutations in this patient (c.180+3A>T and c.1488G>T), we interpret this case as an obvious double-trouble situation. The ClC-1 channel is mainly expressed in the skeletal muscle and only at limited levels in smooth muscle, kidney, heart, liver, and CNS [30, 31]. Since no extra-muscular and especially cardiac manifestations of NDM are reported in other NDM-CLCN1 cohorts so far and given the low number of patients with cardiac arrhythmias in our cohort, a causal relation between *CLCN1* mutation and cardiac manifestations stays unlikely. However, NDM patients must undergo regular cardiac evaluation as the intake of anti-myotonic drugs can unmask latent and potentially life-threatening arrhythmias.

The mean diagnostic delay of this cohort was quite long (16.6 ± 12.2 years), even for a rare disease. Similar results were however reported by Baumann et al. (18 ± 14 years) and Trip et al. (12.0 ± 10.4 years) [21, 24]. From our results, it appears that the longest delay occurred between the onset of symptoms and first EMG performed (12.6 ± 10.7 years). This suggests that patients are often not immediately referred to a neurologist, being misdiagnosed as fibromyalgia or chronic pain syndrome; in other cases, patients with mildly pronounced symptoms, negative family history, normal CK will not seek immediate medical consultation. A normal CK was found in 39.6% of our patients, thus this should not prevent the suspicion of NDM or the referral to a neurologist. In the current German guidelines on NDM, the measurement of CK is a mandatory part of the diagnostic testing and is stated to be no more than 5 × upper normal limits (UNL) for NDM-CLCN1 and often more than twice the UNL for SCN4A-myotonias [11]. In some patients with SCN4A-myotonia, we observed CK elevation up to 1100 U/L. These values may have been influenced by various factors, such as physical activity on the previous day. The EMG detected myotonic runs in 89% of NDM-CLCN1 and 80% of NDM-SCN4A patients without provocative cold test and confirmed to be the most valuable diagnostic tool for the identification of NDM patients and indication of genetic testing.

In our NDM-CLCN1 cohort, the common mutation c.2680C>T (p.Arg894*) was found in 50%. This typical Thomsen *CLCN1* mutation, confirmed in the original Thomsen family from Northern Germany, is also frequently found in Northern Scandinavia, Russia, Denmark and to a lesser extent in The Netherlands, Spain, and Italy [32, 33]. It still has a high prevalence in northern Europe. Other mutations were most frequent in Spain (c.180+3A>T), the Netherlands (c.1238T>G, p.F413C), and Italy (c.501C>G, p.F167L) [25, 34–36]. In our study, no clear genotype–phenotype correlations could be identified, this might be also related to the several different mutations that our patients presented throughout the genes. Some previous functional studies have demonstrated how the location of the mutations in the different channel domains deeply influences the clinical severity and response to therapy producing major differences among patients sharing the same gene defect but different mutations. On the other hand, different phenotypes have also been described in patients with the same mutation, suggesting that modifier genes and environmental condition might influence the clinical features of individual patients [37, 38].

We found 12 novel genetic variants. Although we did not perform functional studies, we included some class 3 mutations that were highly suggestive of being pathogenic due to investigation of family members, indicative clinical symptoms and the evidence of EMG myotonia. Very interestingly, patient 21 displayed the dominant c.3917G>T mutation on both alleles. This change from glycine to valine is known to cause moderate to severe myotonia [39] and this patient displayed earlier and more severe symptoms compared to other affected family members.

The main differential diagnosis of NDM were dystrophic myotonias, with a national biased approach, like myotonic dystrophy type 2 being tested slightly more frequently than myotonic dystrophy type 1 in our sample. Other genes examined in these NDM patients were *CAV3, ATP2A1* (Brody disease) and *HINT1* (hereditary neuromyotonia with axonal neuropathy). Since 2010 next-generation sequencing can be used in the clinical setting in Germany. Therefore, we wanted to examine whether this new sequencing technique reduced the diagnostic delay of NDMs. However, no significant differences were observed for the diagnostic delay in patients diagnosed before and after 2010. Probably because even before 2010 tandem analysis of *CLCN1* and *SCN4A* was commonly conducted.

The effective symptomatic treatment of NDM remains a challenge. At their last follow-up visit, about 50% of our patients (44.7% NDM-CLCN1, 63.6% NDM-SCN4A) were not taking any anti-myotonic therapy. Trivedi et al. reported the use of anti-myotonic medication in 60.6% of patients, Dupré et al. in 41% of recessive (36% with significant improvement), 0% of dominant NDM-CLCN1 patients and 43% of NDM-SCN4A patients. As described by Dupré et al., phenytoin and gabapentin were most effective in patients with recessive CLCN1-myotonia, while mexiletine and carbamazepine were most effective in patients with SCN4A-myotonia [12, 22]. The most frequently used anti-myotonic drugs were carbamazepine, flecainide, mexiletine and lamotrigine in this order of frequency. Flecainide and mexiletine are the drugs of first choice according to the current German guidelines, carbamazepine was more used in the past and lamotrigine more recently after the clinical study by Anderson et al. [11, 16] Patients rated mexiletine, and lamotrigine as the most effective therapies, in accordance with recent RCTs [15, 16], whereas other medications were mainly considered as not satisfactory. This is also confirmed by the high number of tested anti-myotonic drugs (up to 11 different drugs). The good efficacy of phenytoin/gabapentin in recessive CLCN1-myotonia and carbamazepine and mexiletine in SCN4A-myotonia reported by Dupré et al. was, with exception of mexiletine, not observed in our cohort [22]. Our data however, lack information regarding the duration of anti-myotonic drug intake and patients’ compliance, which may impact the evaluation of drug efficacy.

The limitation of our study is its retrospective design. The collection of information has been likely affected by the accuracy of different physicians in record keeping. Despite this, the strength of our study lies in a large number of genetically confirmed patients, and the opportunity of a direct comparison of patients with either a *CLCN1* or a *SCN4A* gene mutation in one single highly specialized neuromuscular center over 25 years.

In summary, this study highlights the clinical, genetic and therapeutic challenges related to the diagnosis and management of NDM patients and unmet needs are the lack of follow-up studies of large cohorts with reliable assessments of disease progression and treatment efficacy.

## Supplementary Information

Below is the link to the electronic supplementary material.

Supplementary file1 (DOCX 31 kb)

## Data Availability

Not applicable.
