# Supplementary material for: Non-dystrophic myotonias: clinical and mutation spectrum of 70 German patients
Source: J Neurol. 2020 Dec 2;268(5):1708–20. doi: 10.1007/s00415-020-10328-1 (PMC8068660; doi:10.1007/s00415-020-10328-1)
Supplement: Supplementary file 1 — Supplementary file1 (DOCX 31 kb) [file 415_2020_10328_MOESM1_ESM.docx]

**Supplementary Table 1: Other tested genes**

| **Gene** | **CLCN1** | **SCN4A** | **Total** |
| --- | --- | --- | --- |
| CNBP | 21 | 6 | 27 |
| DMPK | 18 | 7 | 25 |
| SCN4A | 12 | 0 | 12 |
| CLCN1 | 0 | 11 | 11 |
| CAV3 | 4 | 3 | 7 |
| ATP2A1 | 4 | 3 | 7 |
| HINT1 | 4 | 2 | 6 |
| ACTA1 | 3 | 1 | 4 |
| GAA | 3 | 1 | 4 |
| Cavin-1 | 3 | 1 | 4 |
| CACNA1S | 1 | 3 | 4 |
| AMPD1 | 1 | 1 | 2 |
| FHL1 | 0 | 1 | 1 |
| SMCHD1 | 1 | 0 | 1 |
| D4Z4-Repeat | 1 | 0 | 1 |
| KCNE3 | 0 | 1 | 1 |
| KCNJ2 | 0 | 1 | 1 |
| RYR1 | 0 | 1 | 1 |
